# Enhancing Indigenous health research capacity in northern Ontario through distributed community engaged medical education at NOSM: A qualitative evaluation of the community engagement through research pilot program

**Published:** 2018-03-27

**Authors:** Marion Maar, Lisa Boesch, Sheldon Tobe

**Affiliations:** 1 Faculty of Medicine, Northern Ontario School of Medicine, Ontario, Canada; 2 Office of Research, Northern Ontario School of Medicine, Ontario, Canada; 3 Department of Medicine, Sunnybrook Health Sciences Centre, Sunnybrook Research Institute, University of Toronto, Ontario, Canada

## Abstract

**Background:**

The Community Engagement Through Research (CETR) program matches Indigenous communities interested in exploring their own health research questions with NOSM learners seeking experience in health services research, supervised by faculty experienced in community-based participatory research.

**Methods:**

Qualitative research was conducted using key informant interviews to examine outcomes of the matching of medical students with Indigenous distributed medical education (DME) communities in NOSM’s distributed curriculum, in particular improvements for capacity for Indigenous health research in Northern Ontario.

**Results:**

Interviews showed that community-centred research was appreciated by community, students and faculty and the social accountability aspect was acknowledged. Students and community members found meaning in the immediate applicability of the research to real community problems and felt inspired by it. The challenges that were identified were mainly related to time and resource constraints, including providing sufficient research training for learners, and the time period required for research ethics board approvals.

**Conclusions:**

The program successfully brought together communities interested in conducting their own health research, with medical students interested in learning about and conducting health research with Indigenous communities. It is therefore an example of successful community based participatory research supporting the social accountability mandate. Challenges are mainly administrative in nature. The program has the potential to be scalable and financially sustainable.

## Introduction

The vision of the Northern Ontario School of Medicine (NOSM) is *Innovative Education and Research for a Healthier North*. To accomplish this vision, the School has a commitment to leading edge, distributed, learning-centred, community-engaged education and research. On the education side, the School has engaged over 90 Northern Ontario community sites within its Distributed Community Engaged Learning (DCEL) model.^1^ At NOSM, community engagement is the core framework for achieving distributed medical education (DME) and meeting the School’s Social Accountability mandate which includes community participation in many aspects of the functioning of the School.^2^ As outlined in a key article in this issue,^3^ NOSM community engagement consists of collaboration with the medical community and the community at large starting even before the program admissions stage and extending to the development of medical students’ and residents’ education and training experiences. The NOSM approach is to design, develop, and deliver the DME curriculum in a way that enhances social accountability. The goal of social accountability in practice at NOSM is to meet the health needs of the community served.^4^ This applies equally to health research as it does to medical education. To ensure that NOSM’s social accountability mandate for Northern Ontario is met for education, NOSM faculty and staff collaborate on the development of the curriculum with many groups that represent this population, including Indigenous, Francophone, remote, rural, and underserviced communities. This includes consultation within these communities to understand the health needs through ongoing collaborative workshops, hosting students in Indigenous and non-Indigenous communities, and formal affiliation agreements setting out the roles and functions of community partners.

Collaborating with Indigenous peoples on learning activities designed to positively impact the health of those living in Indigenous communities is one of the aspects of social accountability at NOSM that can be accomplished using distributed community engaged learning. As an example, NOSM students participate in a mandatory DME module where students are placed in pairs for cultural immersion for four weeks in one of over 40 Indigenous communities, ranging from northern semi urban to rural and remote communities.^5^ Development and maintenance of long-term partnerships with the Indigenous communities are assisted by the NOSM Indigenous Affairs unit.^5^

This level of mandatory learning in distributed Indigenous communities with the goal of producing culturally safe physicians is unique to NOSM.^6^ However, the goals for DME in rural and underserved communities are increasingly expanding beyond the production of competent rural doctors. There is a desire for medical schools to better address the health needs of the population on a population level^7^ and evidence is emerging of these added benefits such as community partnerships and community development.^8^

At NOSM, one of the new positive outcomes explored is Indigenous health research. Some communities have expressed interest in collaborating on health research with NOSM students after their placements in Indigenous communities. This interest to collaborate at some of the Indigenous learning sites provided the impetus for the development of the Community Engagement Through Research (CETR) program.

### Community Engagement Through Research (CETR)

CETR is a NOSM program in its third year of development. The overall goal of the program is to build on the existing DME relationships to strengthen research linkages between NOSM and Indigenous communities and to increase capacity for Indigenous health research amongst medical learners, trainees and communities. To accomplish this goal, CETR matches Indigenous distributed education sites where community leaders have expressed interest in exploring health research questions with NOSM learners who seek experience in health services research. Learners are supervised in this endeavor by faculty experienced in collaborative research. Matches are based on learner and community compatibility with respect to their research interest and expertise. The timing of the community portion of the research is typically June to August. Supervising faculty are senior NOSM researchers with expertise in conducting community-based participatory research (CBPR) with Indigenous communities. Learners are medical students (mostly those who have completed their first year of studies) or residents. Health research projects are varied and may be based on either data analysis of health databases such as local health records or administrative data sets like those housed at the Institute for Clinical and Evaluative Sciences (ICES). Qualitative research projects are also possible. The most important aspect of the CETR approach is that the project is community driven, instead of investigator driven.

NOSM faculty researchers’ supervision tasks include mentorship for (1) institutional research ethics review, (2) the community involvement according to the principles of community based participatory research, and (3) knowledge translation. In keeping with NOSM’s social accountability mandate, the underlying philosophy of the program is to facilitate health research that is initiated by the Indigenous community to assist the community to answer research questions that it has identified as important. This ensures that the research is culturally appropriate and community controlled.^9^ For the knowledge translation aspect, the learner is expected to write up the research findings after presenting the data to the community and community members are invited to collaborate on academic dissemination.^10^ In cases where the community is uncomfortable with the sharing of the findings, the community is in control of the data under the principles of Ownership, Control, Access, and Possession (OCAP) and dissemination is based on a negotiated consensus.

Our current research examines some of the successes and challenges to the synergistic relationship for socially accountable learning and research enabled by NOSM DME collaboration with Indigenous community sites. To accomplish this, we conducted qualitative research to elicit the perspectives of Indigenous community leads, students, and faculty who have participated in the program. We also discuss supports and future development needs for this CETR program.

## Methods

### Study design

We undertook qualitative research to determine if this community engaged research program, that matches medical students with Indigenous DME communities in NOSM’s distributed curriculum, can enhance capacity for Indigenous health research in Northern Ontario. At the time of the study, the CETR project had taken place in four Indigenous communities. A list of the community research projects undertaken by the medical students are presented in Table 1.

**Table 1:** Community research projects

Year	Project
2014, 2015	Obesity Among Indigenous Youth
2015, 2016	Experience of Aboriginal Patients Who Must Relocate to Sioux Lookout for Hemodialysis Services
2015	The [medical student] researcher’s guide to chart review: challenges and ‘pearls’ for designing a study that involves the secondary use of Aboriginal patient data
2016	Commercial Tobacco Use: Learning how to provide Post Study Knowledge Translation of Value to the Community

All students were supervised by NOSM faculty experienced in long-term community-based participatory research in partnership with Indigenous communities. The faculty members supported the students with community engagement practices and dialogue by attending community meetings and supervising all phases of the data collection, analysis and knowledge translation.

Participants were invited to contribute through semi-structured telephone interviews and asked open-ended questions to discuss their perspectives and experiences about the program. Three different, but complementary, interview guides were developed: 1) Community contacts were invited to comment on how they viewed the research and whether they felt that the program helped their community to be more research aware and research friendly; 2) Medical students were invited to share their experiences of the research process and to determine what impact the program has had on their ability to conduct health research; and 3) Faculty supervisors at the main and the distributed sites were invited to share their perspectives of the challenges and opportunities related to supervising students who conduct the research.

### Participants

All student researchers, community contacts and supervising faculty involved in the program over the past three years were invited via email to participate in this research - a total of 14 including six students, five community contacts, and three faculty members. Eleven of these people agreed to participate within our time frames. One of our author group (LB) conducted all of the telephone interviews.

### Data analysis

All interviews were audio recorded and transcribed verbatim by one researcher (LB). Participants were randomly assigned a letter as a pseudonym to protect their identity. QSR International’s NVivo 9 qualitative data analysis software was used for coding and analysis of the transcripts. We used a primarily deductive approach to coding when developing the themes to identify perceived benefits and challenges to conducting CETR projects at the Indigenous DME community sites and required supports and resources. However, we also integrated inductive coding within these major categories. As such we identified emerging themes based on the research team’s collective experience in conducting CBPR in collaboration with Indigenous communities. Draft themes were reviewed within the research team and finalized during discussions at several research meetings.

### Ethics

Research Ethics approval for the research protocol was obtained from Laurentian University (REB #6009524). This evaluation research of the CETR program was also approved by the First Nations community collaborators in line with the Chapter 9 of the Tri Council Policy Statement.^11^ Community partners reviewed and supported the analysis of the findings.

## Results

A total of 11 people participated in the interviews. Table 2 shows a summary of participants from each of the invited groups.

**Table 2:** Number and description of participants in interviews

Group	# of Participants
Medical Students	6
Community Contacts	3
Faculty Members	2
**Total**	**11**

The analysis provided several themes under each of the major categories of the perceived benefits and challenges related to CETR projects at the Indigenous DME community sites.

### Benefits of CETR

Our thematic analysis shows that project benefits to community and students were perceived within three main areas: a) meeting community information needs; b) understanding the importance of developing trust-based Indigenous research relationships; and c) practicing CBPR principles.

#### Meeting community information needs

Meeting the needs of the community is the central value of the CETR program. Interviews showed that the value of community-centred research is appreciated by community, students and faculty. Community contacts stressed that their staff’s focus on services made it difficult to see research projects to completion without a collaborating researcher:

…it’s allowed us to kind of just revisit those projects and surveys and evaluations that we sort of just left to the wayside […] not purposely but just by the nature of the needs of the community that keep us from saying ‘okay well we’re going to work on research for the next two months.’ Like we just don’t have the capacity or the resources to do that. […] It’s almost like we need – we don’t have a research department or we don’t have anyone specific that’s assigned to looking at all of this and following up with all of this, so certainly I think that helps us. [W., community member]

Similarly, another community member explained that data collection had been ongoing in the organization to meet their information needs, however data analysis had not been conducted:

“we’ve been collecting this data for a number of years […] [and thought] it would be interesting to see what we could do with this data […] and possibly compare it to other First Nations or other schools across the region. So that kind of I guess ignited the discussion, so that’s when […] we started research with [the medical student]. […] It was a good experience and when the results did come back, our community was really excited. [N., community member]

I think all those initiatives that we’ve been working on over the last few years here we wanted to kind of validate it and to see what the outcomes were. So that was the biggest [success], it was validation of some of the programs that we were hoping for but also to give us some kind of recommendations as to what we can also do to improve. [N., community member]

Students found meaning in the immediate applicability of the research to real community problems and felt inspired by it.

And also one of the big successes was that I felt that we concentrated on a research question that was useful to the community. It wasn’t just a medical student idea or token of research to get the mark. It was in fact something that was really important to the community so that made me want to work hard at it and make sure everything was done in a rigorous manner. [P., medical student]

One faculty member discussed the innovation of exploring the applicability of large administrative databases to address local First Nations issues using CBPR.

I think the intentions are good to harness the resource potential that ICES has and to liaise with First Nations communities about what research needs they may have. [C., faculty member]

However, the use of data sets that already existed at the community level and the use of those data to answer community research questions was seen as important to community empowerment and social accountability through research.

…the question that [the health services staff] had was, could their data set give them any information on the effectiveness of their own program within the community. We were able to do a comparison with Stats Canada, we were able to provide the manpower with students over two different summers, to do the legwork…so I think that was a real win for the community. [Q., faculty member]

#### Understanding the importance of building trust-based Indigenous research relationships

Many Indigenous communities have had previous negative experiences with health research and are hesitant to engage in projects unless they can trust the collaborators.

…from previous research that was done in the community, it seemed to be more negative but I think we’re starting to turn the table and we’re seeing positive results, but also positive relationships with our researchers so that building that trust and making sure that that information is going to be beneficial to our First Nation. [N., community member]

…when I speak about research with the community, I want it to be something that [community members] feel comfortable with and they kind of can relate to it […] Right now I know we’ve got a lot of work to do in terms of righting the wrongs of the past and being surveyed to death and being asked and being taken away, like all this information and where does it go and how does it benefit us. And I think that’s why people are very skeptical and they’re guarded and I think I’d like to be part of that change to ensure that we can turn that around into a positive thing and say well this is what we’re doing now, so people can become comfortable with that and say “Oh yeah the health center’s doing research, that’s great! [W., community member]

Working on the CETR projects, students began to experience firsthand the significance of establishing long-term working relationships in Indigenous communities.

When you have a pre-existing relationship with the community, then really good things can happen. And things can happen more quickly because you have been a part of research and interactions with First Nations communities. … When there’s not an established relationship then there’s more of a hesitancy. Things just don’t work as quickly or as smoothly… I had that previous relationship with the First Nation and the administration supported the research that they were hoping to do with open arms. I really felt a part of the research team rather than kind of like as an outsider. So I felt part of the team and useful. [P., medical student]

Students learned that the community relationship that they personally began to value also had positive effects on the research collaboration process.

Working within communities that you’re familiar with is great. Or being able to develop a relationship with the community I think is a big benefit as well and that’s something I definitely took away. Those relationships that I built in the community, I really value. [D., medical student]

Several spoke about how they genuinely enjoyed the relationship building process.

I enjoyed being able to spend some time in the community itself and get to know the people who I was working with and who were involved in the study. [S., medical student]

Finally, the positive interactions motivated the participating student researchers to collaborate in future research projects with Indigenous people.

When I think back to the project I’m not thinking about the numbers or the literature. I’m thinking about the people that I met. … I do hope that [this program] continues just because I think it’s nice to continue the relationships that we’ve established in the Aboriginal communities *that we’ve been part of. [D., medical student]*

#### Practicing CBPR principles

The principles for CETR are strongly rooted in CBPR, because a collaborative approach is the most important aspect of research with Indigenous communities supported by the literature as well as the perspective of the community participants in this study. The importance of benefit from the perceptive of the community was a theme stressed by community members:

Well we’re very careful in the research we conduct. It has to be valuable research that’s for the benefit of our community and not sole benefit of the researcher. And we look at it as is it going to improve the quality of life of our community members. And if it’s not going to be for the benefit of our [community], you know, we’re very reluctant to get involved with research because we’ve seen too many in the past where research was done and information that was given to researchers never come back to the community. So we try to make sure the community is engaged and that we know it’s going to be beneficial and they’re a trustworthy research organization. [N., community member]

Examples of benefit were articulated in various forms.

This is just one example of how we’re engaging with NOSM to look at research. How does [research] appeal to us as an Indigenous community so that we feel like we’re being engaged, and we feel like we’re being heard, and we feel like we’re being supported. And more importantly the knowledge translation must be there, to the people who it impacts the most and [the assurance] that it’s going to make a difference to them. You know and part of [the engagement in research] is capacity building too, right? [W., community member]

One student perceived the CBPR process as a major factor in the success of the summer research.

I don’t want to speak for the community but I got the sense that [my community collaborators] really enjoy having control over the project and knowing that they could have a student researcher with similar values and similar interests come and work on their issue - like the problem that they’ve identified. So just personally I think they really enjoyed that … my personal and professional values were very much in tune with what the community wanted to do, as well as their values and their culture. So there were a lot of similarities. More similarities than differences which I think helped make this partnership so strong and smooth. [V., medical student]

Additional benefits for student learning as potential future physician researchers were that they experienced the importance of applying CBPR principles in improving Indigenous health and began to value this practical experience.

The community-based aspect of it I think is amazing, and I think that it’s a real strength as you do feel like the research you’re doing is actually valuable because someone has either requested the information and it’s quite obvious how it can lend itself towards hopefully improving the health of a group of people in some small way. [S., medical student]

The other big part that I feel is really beneficial for me personally was kind of living out the principles of community based participatory research. There’s a lot that – I’ve read about it, heard about it, but I actually had an opportunity, you know, to jump in my car and go to the community and meet the people and live with one of the community members and participate in their social activities and get them to know me and talk about research, talk about life, and establish those strong partnerships. So that was really, really neat because not only did it teach me about research, it also taught me about the Aboriginal culture and working with Aboriginal people. [V., medical student]

Looking at social accountability from the faculty perspective, the CBPR aspect was very valuable in establishing or enhancing relationships with communities for future research.

When we presented to [one community] the results of the study, there was so much excitement. The health portfolios were there, the education portfolios were there, and they said this is great! We would like to be able to access a “NOSM researcher in a box” at any time in the future. (laughter) That was their quote! And it generated tremendous goodwill that was already present in that community, but even more goodwill towards NOSM and it also put research in a good light in the community and really reinforced the benefits of community-based participatory research. [Q., faculty member]

### Challenges of CETR

The challenges identified were mainly time and resource constraints, including time commitment required to navigate Research Ethics Board (REB) review process and ensure ongoing commitment to CBPR while restrained by a short timeframe. Additional themes revolved around the highly variable background preparations of students and communities related to health research.

#### Navigating REB process

Navigating the REB process for Indigenous CBPR can be time consuming at the university level. Indigenous research may be under greater scrutiny since deliberations as to the risk of the research is difficult to ascertain for REBs. Additional research review at the community level and formal letters of community support are often required. Related to the question on challenges, one participant observed:

…ethics is always going to be a big one and I *mean rightfully so. [D., medical student]*

It is perhaps not surprising that the process was seen as quite difficult by the student researchers, who, in order to meet their learning goals had to lead the REB application process. The timing of the CETR is during the summer months, the same time when faculty who populate the REB may be conducting research away from the university or take vacation, which slows down the review process. Eager to conduct ethical research, students did appreciate the importance of diligent ethics review. In spite of their good attitudes, the lengthy process frustrated students.

So the ethics process, it was somewhat cumbersome which is … it’s meant to be for extended reasons. The one frustrating part about it is that they couldn’t find anybody to review our proposal because it was summer time and basically people were on vacation and for the longest time it just kind of sat at the ethics office awaiting a reviewer. So after they found someone, it took [only] a couple of days… [V., medical student]

Another student shared their comparable experience:

…the actual making of the REB application was fine because I had to write a protocol anyways. So I had to go through all those steps and all that work and really think about the ethics of the study and how we’re going to make it as ethical as possible. So that was fine and it was a really good exercise … So that part of the process was good, but then once I submitted it, it was in the summer and it just took – like it took so long for me to hear back. [H., medical student]

It is however important to stress that students who have had experience with navigating REB applications planned more effectively and thus were less distressed and their project less affected by REB delays:

Time is something that I knew [the ethics process] would take, so that’s a bit of a challenge because you need to set aside two, three months to have that research ethics process go through and if it’s sent back you need to review and revise. So that’s a challenge and then certainly doing two ethics applications is also a challenge. It’s certainly not insurmountable because we did it, however that’s something that needs to be taken into account. [P., medical student]

Due to the time required to obtain REB approval, faculty at times had to complete research that was still outstanding and as research timetables had to be adjusted due to the delays. The additional work fell to the supervising faculty.

The interviews that I did [this winter], were [part of] summer students’ projects that didn’t happen in the summer because of ethics. So I needed to go out and do both sets of interviews on the student’s behalf because they weren’t able to finish the projects in the summer. [Q., faculty member]

#### Ensuring ongoing commitment to CBPR principles

While the commitment to ethical Indigenous research which is based on the CBPR principles was seen as a strength and benefit, it also posed challenges. CBPR takes time and long-term commitment, with a primary focus on community benefit as defined by the community, not university timetables. Health research conducted in collaboration with Indigenous communities requires not only experience but also time commitment to build meaningful long-term relationships. Faculty also stressed the need for making time not only to develop the relationships but also ongoing community involvement in decision making and data analysis.

Any kind of relationship needs to develop over time, be well informed and well resourced… Research needs in First Nations are complex and …so it’s not as simple as getting data from ICES to give a number or percentage to people. Everything has to be interpreted both from the medical perspective and from the community perspective. [C., faculty member]

After the research is completed, students are often interested in academic dissemination, which also requires community collaboration in CBPR. This is challenging once the new school year begins.

I have written most of the article but we had wanted community input and we, again, through one of the meetings that we had, there was one community member that was pretty interested in getting involved. So we sent the article to the community for review and then I haven’t been great at following up with it just because my schedule is pretty busy and yeah, so the article basically is still unfinished. [D., medical student]

Finally, the nature of crowded and heavy medical school curricula makes it difficult for students to manage the demands of CBPR.

A huge challenge is the medical student’s schedule and having sufficient time to do a research project and taking it from beginning, asking the question, getting approval through the community with Band Council Resolution, ethics, generating the methodology, doing the study, doing the background systematic review to prepare the introduction for the project, presenting it to the community, summarizing it and finally presenting it and doing the knowledge translation all takes a minimum of a year. And a student just doesn’t have that time especially for a summer program. So that was a challenge to try to fit that schedule to a student and not have them have a bad research experience because they couldn’t get the project completed. [Q., faculty member]

#### Responding to the variability in researcher and community readiness

Participants relayed considerable variability in experience and allocable resources at the level of the community and researchers. Faculty recognized that there is considerable variability in student preparedness with research in general and Indigenous research in particular, and that this challenge must be mitigated by an experienced, supervising faculty researcher.

Taking the most vulnerable population and pairing them up with the most inexperienced learners/researchers and hoping for the best…that doesn’t work…You can’t match up those two parties without an intermediary who’s got good relations with the population being studied and is funded or resourced to closely supervise the learner. [C., faculty member]

This past quote also underscores the importance of connecting the student researchers with established researchers who are willing and able to mentor the student researchers in a meaningful way. Less experienced student researchers require more supervision, particularly when matched with communities with little research experience. The risk is that the student might unintentionally breach ethics protocols or the OCAP rules leading to loss of trust between the community and the medical school, potentially with wider negative fallout for everyone. Nonetheless, it is important to remember that some Indigenous communities are quite empowered when it comes to their community conducting and monitoring research initiatives. In these cases, detailed discussions on the summer work flow and planning of activities will be more important than the more basic dialogue on CBPR values and principles.

Medical students as well typically have varied research experience. Some may require special training in quantitative or qualitative research methods to conduct the research, which needs to be done prior to collecting data, particularly when working with qualitative data from interviews. The following quote demonstrates a lost opportunity:

I had a student who was a very nice student, very keen… the student had no experience with how to do a qualitative study, no experience how to do a qualitative interview… I felt bad for the student that s/he was unprepared and that despite me spending half a day with him/her talking about interviewing people and as I’ve done interviews like this in exactly the same setting… [C., faculty member]

## Discussion

Research can be a sensitive topic in Indigenous communities and there is a large body of literature documenting the harms many communities have experienced by investigator-driven research.^5,9-11^ For Indigenous research projects to be successfully implemented and executed, it is vitally important to develop and maintain positive trust based relationships between communities and researchers and this sentiment was strongly expressed in our community interviews.^12-14^ Our research shows that the CETR program has the potential to maintain positive trust based relationships between medical learners and Indigenous communities. Furthermore, medical students experienced the importance of relationship building in Indigenous research. Yet, to ensure the sustainability of the CETR program several strategies are required.

### Formalize the supervisory relationship between faculty and student

The challenges encountered are related to the somewhat unpredictable nature of research, which requires oversight by an experienced researcher to manage the project within certain timelines. During the pilot phase, CETR was implemented mostly ad hoc. In order to develop a sustainable program, the selection of students and matching with communities should become more formalized. For example, students and communities could apply before the winter term, and the faculty member could be more formally assigned to supervise the student and collaborate with the community. The faculty member could evaluate the research skills that the student has and which areas need to be developed. The student could begin some organizational work and participate in meetings and perhaps even visit the community if the student did not attend the community during a disseminated medical education module. Ideally there would be an existing research relationship between the faculty member and the community. This way, culturally safe research is more likely ensured as the program becomes established and this mitigates the risk of having an inexperienced student researcher taking on an Indigenous health research project.

Further recruitment of more faculty members to the CETR program would ensure that research questions are well formulated by the community and refined or followed over several years and with the involvement of several students over multiple years.

From the perspective of teaching research methods to students, training prior to the project, for example in qualitative research interviewing, would be very helpful. Mentoring with an appropriate researcher for longer term is necessary for those students who do not have significant research experience. This model would also be beneficial as data collection training can begin early and be closely monitored.

### Initiate ethics application early

Health research often takes several months to review by an REB and if the research is delayed for the student, the supervisor is left to ensure that the project is completed according to the CBPR principles. Multiple ethics review requirements from academic and community or hospital REBs also pose a challenge to CBPR. For the success of CETR it is necessary to start the REB process several months before the research is anticipated to begin. This requires a process to involve the student prior to the beginning of the official research internship.

### Develop hand-over guidelines for multi-year projects

The time for a single medical student to be involved at each community site is typically one summer, however due to the time and resources challenges, a multi-year approach is often required. When a new student is recruited, handover should occur smoothly. Community staff stressed that they are busy providing services and research is not something that they can make a priority. Therefore, any support from the researcher to keep the project on track through all of the phases in a way that is transparent and easy to follow is welcomed by communities.

An uncomplicated, online project work plan outlining the various tasks associated with the phases of research, such as ethics applications, data types and data collection, analysis and knowledge translation accessible to the community, faculty and students could help to alleviate this issue. It would also be a good tool to monitor progress and manage expectations.

### Identify sustainable funding

The summer students were initially funded by seed money from the Canadian Heart and Stroke Foundation which provided sufficient funds for the three-year pilot. When one community was contemplating a research project for the following year and heard that it might not be possible as the funding had been exhausted, they immediately offered to provide the funds (typically $6,000-$8,000 CDN for the summer student including travel). The community perceived the value of the program of the student and supervisor was worth this modest investment. Other communities have expressed interest in this model making this project potentially sustainable with community funding. However, not all communities are able to afford to pay student salaries, therefore it is important to continue to explore governmental and non-governmental agency funding for this program to ensure financial barriers can be addressed if necessary.

### Track and evaluate research output

The six summer research projects led to four abstracts presented at NOSM’s Northern Health Research Conference (see Table 1). Results from one of the qualitative projects is scheduled to be presented to the Health Directors annual meeting in 2017 for a large provincial treaty organization, but at the community’s request there will be no publication. Another project that was delayed due to ethics approval will continue this coming summer. With community approvals in place, two manuscripts are currently in preparation. It is important to track academic outcomes as well as community impact and obtain feedback from communities if the research outcomes met their expectations and information needs.

### Limitations

The communities, students, and faculty involved were all highly interested in furthering Indigenous research. Communities and faculty had previous Indigenous health research experience and NOSM students had completed one month of cultural immersion curriculum. We expect that some Indigenous research experience is an essential prerequisite to the success of this program and less research experienced or culturally trained students and faculties would likely have much less satisfactory results. While we had good participation rates (11/14), some of the interviews were conducted quite a few months after the project. Some of the participants’ recollections might have been richer and more nuanced if the interviews could have been conducted during their research or immediately at the end of their project.

### Conclusion

The Community Engagement Through Research (CETR) pilot program successfully brought together communities interested in conducting their own health research, with medical students interested in learning about and conducting health research with Indigenous communities. It is therefore an example of successful community based participatory research. There were challenges with the program, especially matching communities with medical students early enough in the school year to allow refinement of the question, community approval for the work, and a successful application for research ethics.

NOSM’s mandatory DME within Indigenous cultural and clinical teaching sites has created long term partnerships with Indigenous communities. These maturing relationships between Indigenous communities and the School have become the foundation for CETR, an innovative approach to enhancing learner and community readiness for Indigenous health research. Our research shows that there is clear indication that the CETR program is seen as beneficial by communities and students, and identified the support for faculty, students and community needed to make the program sustainable. Similar projects could be explored at the DME teaching sites at other medical schools.

## References

[1] Strasser RP Community engagement: a key to successful rural clinical education. Rural Remote Health. 2010 9;10(3):1543. 20815653

[2] Strasser R, Lanphear J The Northern Ontario School of Medicine: responding to the needs of the people and communities of Northern Ontario. Educ Health Abingdon Engl. 2008 12;21(3):212. 19967640

[3] Strasser R, Hogenbirk J, Jacklin K, Maar M, Hudson G, Warry W, Cheu H, Dubé T, Carson D Community engagement: A central feature of NOSM’s socially accountable distributed medical education. CMEJ. 2018;9(1):33-43. PMC6104326 30140333

[4] Strasser RP, Lanphear JH, McCready WG, Topps MH, Hunt DD, Matte MC Canada’s new medical school: the Northern Ontario School of Medicine: social accountability through distributed community engaged learning. Acad Med. 2009;84(10):1459–64. 19881443 10.1097/ACM.0b013e3181b6c5d7

[5] Jacklin K, Strasser R, Peltier I From the community to the classroom: the Aboriginal health curriculum at the Northern Ontario School of Medicine. Can J Rural Med. 2014;19(4):143–50. 25291039

[6] Hudson G, Maar M Faculty analysis of distributed medical education in Northern Canadian Aboriginal communities. Rural Remote Health. 2014;14(4):2664. 25277126

[7] Schofield A, Bourgeois D Socially Responsible Medical Education: Innovations and Challenges in a Minority Setting. Med Educ. 2010;44(3):263-71. 20444057 10.1111/j.1365-2923.2009.03573.x

[8] Lovato C, Bates J, Hanlon N, Snadden D Evaluating Distributed Medical Education: What Are the Community’s Expectations? Med Educ. 2009;43(5):457-61. 19422493 10.1111/j.1365-2923.2009.03357.x

[9] Smylie J, Kaplan-Myrth N, Tait C, et al Health sciences research and Aboriginal communities: pathway or pitfall? J Obstet Gynaecol Can. 2004 3;26(3):211–6. 15016333 10.1016/s1701-2163(16)30259-6

[10] Maar MA, Lightfoot NE, Sutherland ME, et al Thinking outside the box: Aboriginal people’s suggestions for conducting health studies with Aboriginal communities. Public Health. 2011 11;125(11):747–53. 22093920 10.1016/j.puhe.2011.08.006

[11] Canadian Institutes of Health Research, Natural Sciences and Engineering Research Council of Canada, Social Sciences and Humanities Research Council of Canada Tri-Council Policy Statement. Ethical Conduct for Research Involving Humans. CIHR, NSERC, SSHRC of Canada, 2014 [Internet]. Available at: http://www.pre.ethics.gc.ca/pdf/eng/tcps2-2014/TCPS_2_FINAL_Web.pdf [Accessed May 29, 2017].

[12] Lightfoot N, Strasser R, Maar M, Jacklin K Challenges and rewards of health research in northern, rural, and remote communities. Ann Epidemiol. 2008 6;18(6):507–14. 18261925 10.1016/j.annepidem.2007.11.016

[13] Maar M, Yeates K, Barron M, et al I-RREACH: an engagement and assessment tool for improving implementation readiness of researchers, organizations and communities in complex interventions. Implement Sci. 2015;10:64. 25935849 10.1186/s13012-015-0257-6 PMC4424962

[14] Manitowabi D, Maar M Applying Indigenous Health Community-Based Participatory Research. Toronto: McGregor and Johnston, 2017 In press.

